# Supplementary figures and images for: ALKBH1 activity in vitro and human cell lines by isotope dilution mass spectrometry
Source: PLoS One. 2026 Apr 6;21(4):e0337155. doi: 10.1371/journal.pone.0337155 (PMC13052853; doi:10.1371/journal.pone.0337155)

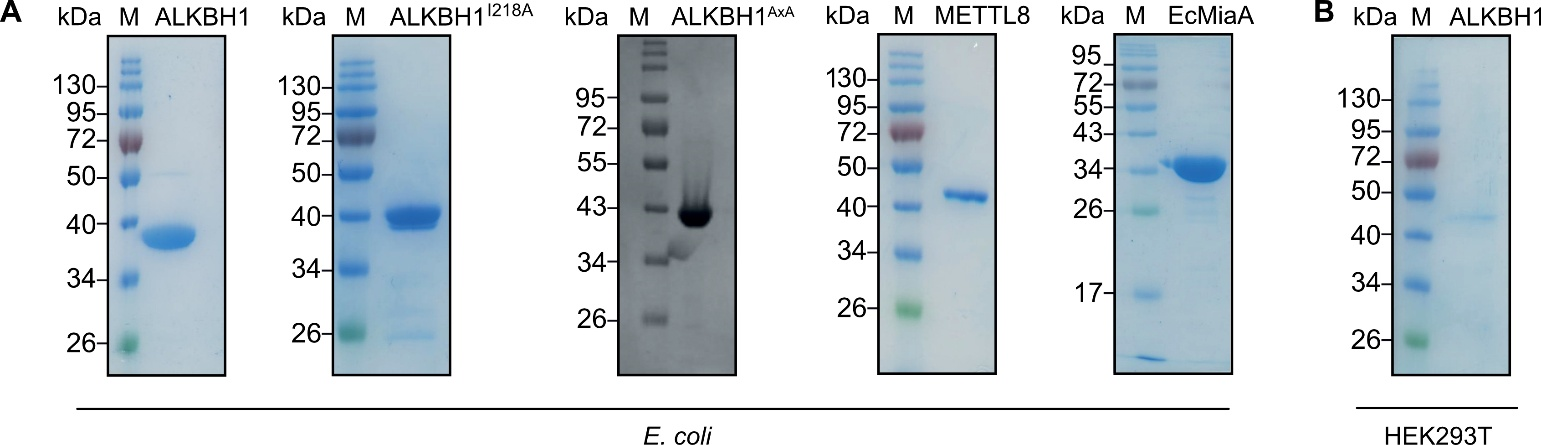

Supplement: S1 Fig — Human ALKBH1 and ALKBH1I218A, ALKBH1AxA, METTL8 and E. coli MiaA were overexpressed and purified from E. coli. B. Human ALKBH1 was overexpressed and purified from HEK293T cells. (TIF) [file pone.0337155.s001.tif]

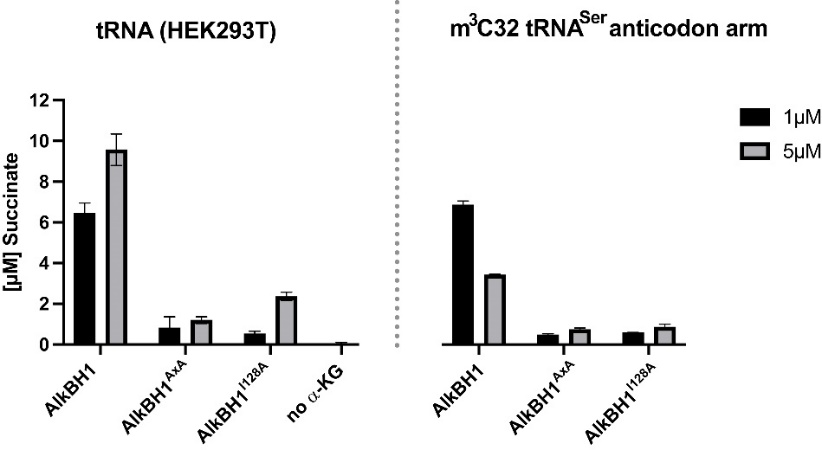

Supplement: S2 Fig — Activity of Fe(II)/α-KG dependent dioxygenases result in the conversion of α-KG to succinate, which in the assay is first converted to ATP that is finally measured by a luciferase/luciferin reaction. The light generated correlates to the amounts of succinate produced by the dioxygenases as a measure for the dioxygenase activity. As substrates either total tRNA extracted from HEK293T cells, as well as a synthetic RNA oligonucleotide as mimic for the mitochondrial tRNASer anticodon arm, with the single m3C32 modification were used. Both were incubated either with 1 μM or 5 μM of wildtype ALKBH1, ALKBH1I218, which cannot undergo auto hydroxylation, or inactive ALKBH1AxA as a control. This shows that the assay is also sensitive to auto-hydroxylation, since overall lower amounts of succinate are generated when ALKBH1I218 is added to total tRNAs, compared to the wild type (left panel). Moreover, the synthetic tRNA-anticodon arm mimic seems not to be a suitable substrate for ALKBH1, since no changes in succinate levels can be observed when ALKBH1I218, which cannot undergo auto hydroxylation was added (right panel). Without addition of α-KG to ALKBH1 no signal is observed. (TIF) [file pone.0337155.s002.tif]

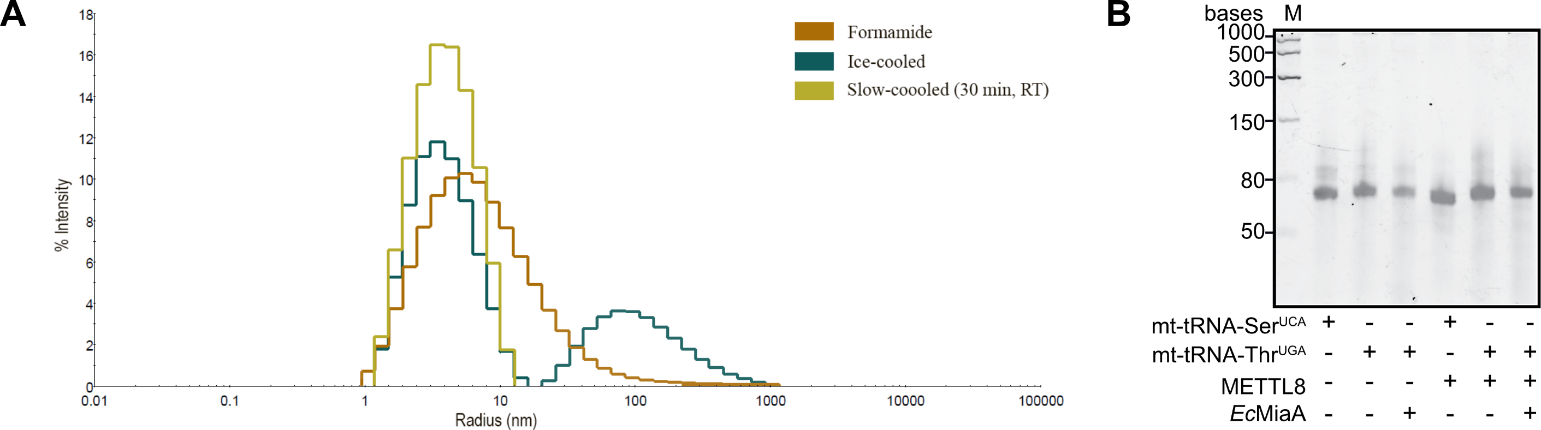

Supplement: S3 Fig — A) Dynamic light scattering (DLS) analysis of folding of in vitro transcribed mt-tRNA-ThrUGU. Denaturing by heat and slow cooling results in monodispersed sample, indicating homogenous and correct folding. Heating followed by rapid cooling on ice leads to aggregation that can be seen by polydispersed distribution. Polydispersity of the sample is also increased by addition of formamide. B) Urea-PAGE (8%) analysis of in vitro transcribed RNA of mt-tRNAs. Both tRNAs were treated with METTL8. Mt-tRNA-SerUCA was first treated with EcMiaA. (TIF) [file pone.0337155.s003.tif]

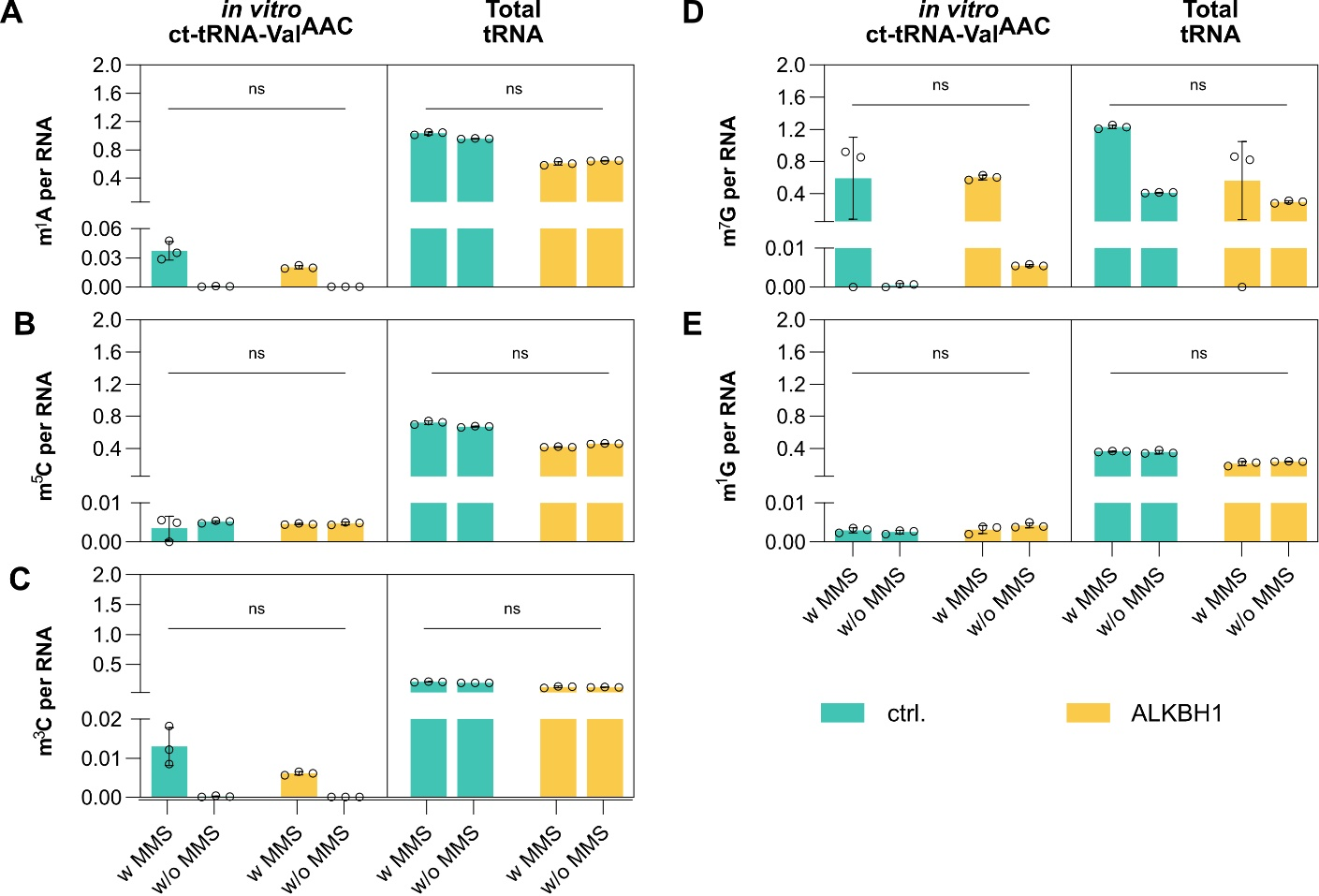

Supplement: S4 Fig — A – E. Absolute levels of m1A (A), m5C (B), m3C (C), m7G (D) and m1G (E) in in vitro transcribed ct-tRNA-ValAAC with and without MMS treatment (w/o MMS). Data represents n = 3 biological replicates. Error bars in graph represent the ± mean S.E. of three biological replicates. p-value (p > 0.05 = ns, p ≤ 0.05 = *, p ≤ 0.01 = **, p ≤ 0.001 = *** and p ≤ 0.0001 = ****) mentioned in the text is calculated by one-way ANOVA and indicates significant differences in medians. (TIF) [file pone.0337155.s004.tif]

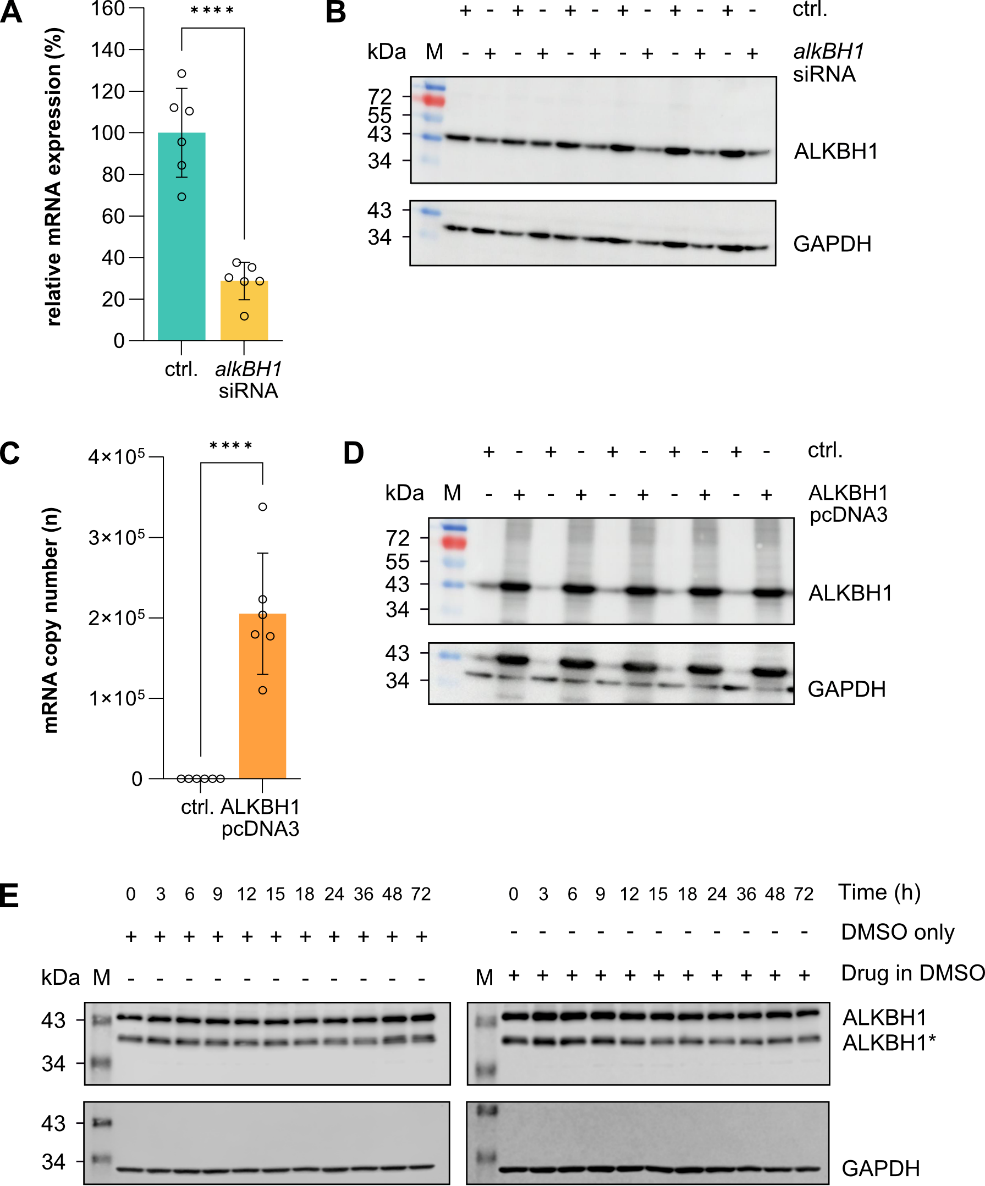

Supplement: S5 Fig — Analysis of in vitro transcribed tRNAs. A) Dynamic light scattering (DLS) analysis of folding of in vitro transcribed mt-tRNA-ThrUGU. Denaturing by heat and slow cooling results in monodispersed sample, indicating homogenous and correct folding. Heating followed by rapid cooling on ice leads to aggregation that can be seen by polydispersed distribution. Polydispersity of the sample is also increased by addition of formamide. B) Urea-PAGE (8%) analysis of in vitro transcribed RNA of mt-tRNAs. Both tRNAs were treated with METTL8. Mt-tRNA-SerUCA was first treated with EcMiaA. (TIF) [file pone.0337155.s005.tif]

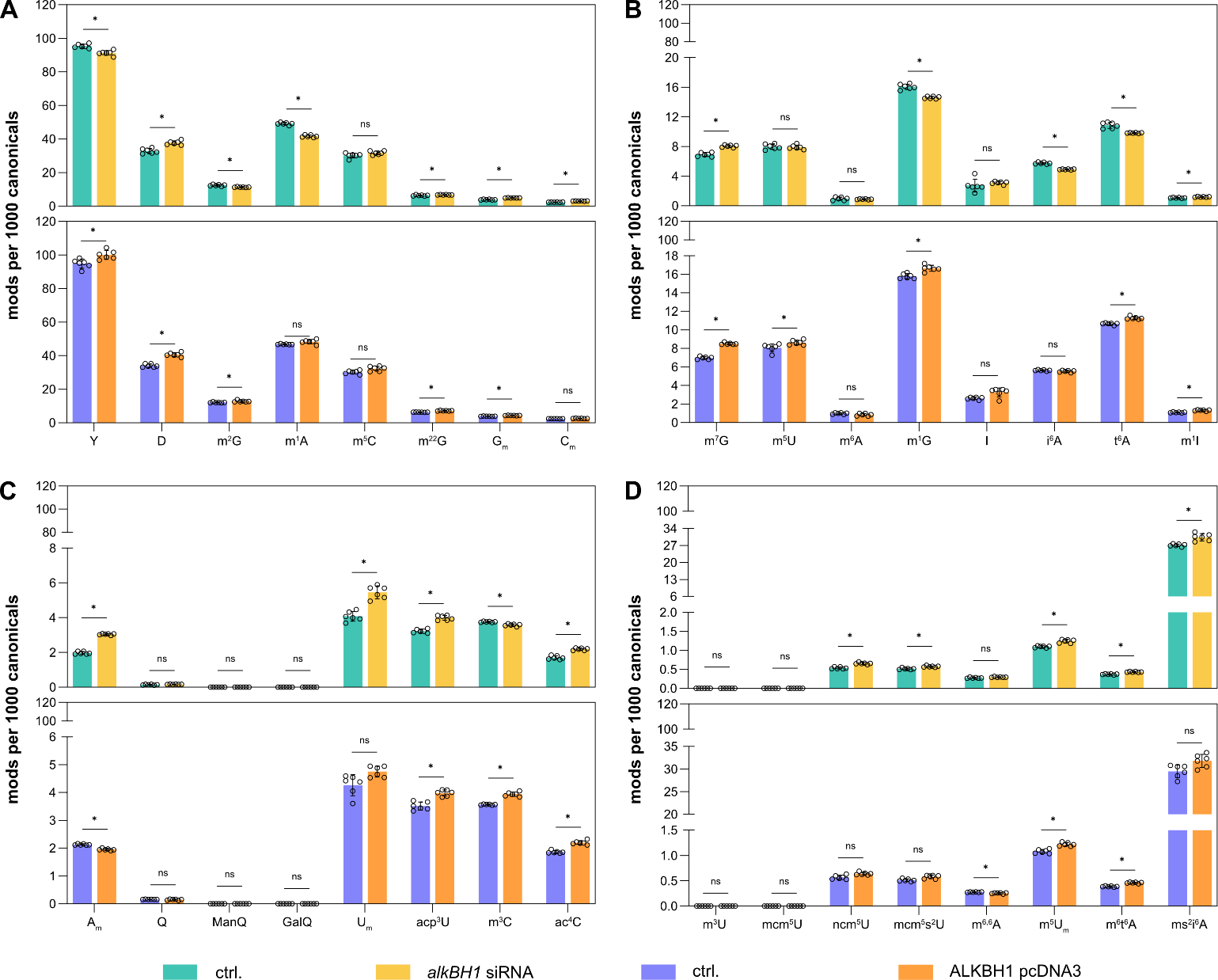

Supplement: S6 Fig — A-D. LC-MS/MS analysis with absolute levels for Y, D, m2G, m1A, m5C, m22G, Gm and Cm (A). Absolute levels for m7G, m5U, m6A, m1G, I, i6A, t6A and m1I (B). Absolute levels for Am, Q, ManQ, GalQ, Um, acp3U, m3C and ac4C (C). Absolute levels for m3U, mcm5U, ncm5U, mcm5s2U, m6,6A, m5Um, m6t6A and ms2i6A (D). Error bars in the graph represent the ± mean S.E. of six biological replicates. p-value mentioned in the text is calculated by unpaired t-test with no correction and indicates significant differences in median. (TIF) [file pone.0337155.s006.tif]

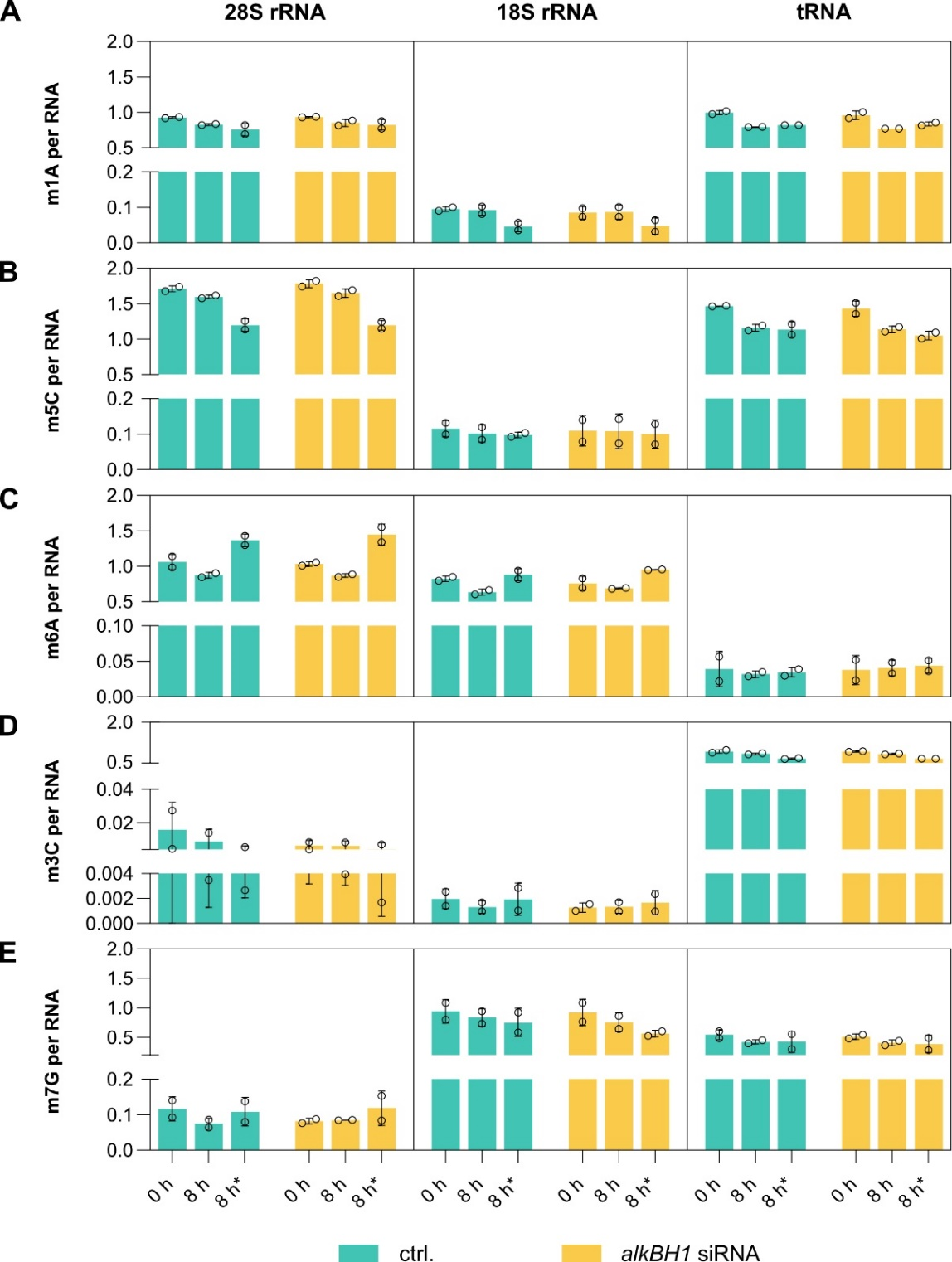

Supplement: S7 Fig — A-E. Absolute levels for m1A (A), m5C (B), m6A (C), m3C (D) and m7G (E) per respective RNA molecule (left column: 28S rRNA; middle column: 18S rRNA; right column: tRNA). Each boxplot is divided into three bars: the left column shows the old, unlabeled nucleosides before medium change (t = 0 h, 64 h after seeding), the middle bar shows the old, unlabeled nucleosides after incubation with stable isotope-labeled medium (t = 8 h, 72 h after seeding), and the right bar shows the new, labeled nucleosides after 8 h* of incubation. At each time point, RNA was isolated from siRNAs targeting ALKBH1 (Negative control “ctrl”: scrambled siRNA). All values are derived from n = 2 biological replicates. (TIF) [file pone.0337155.s007.tif]

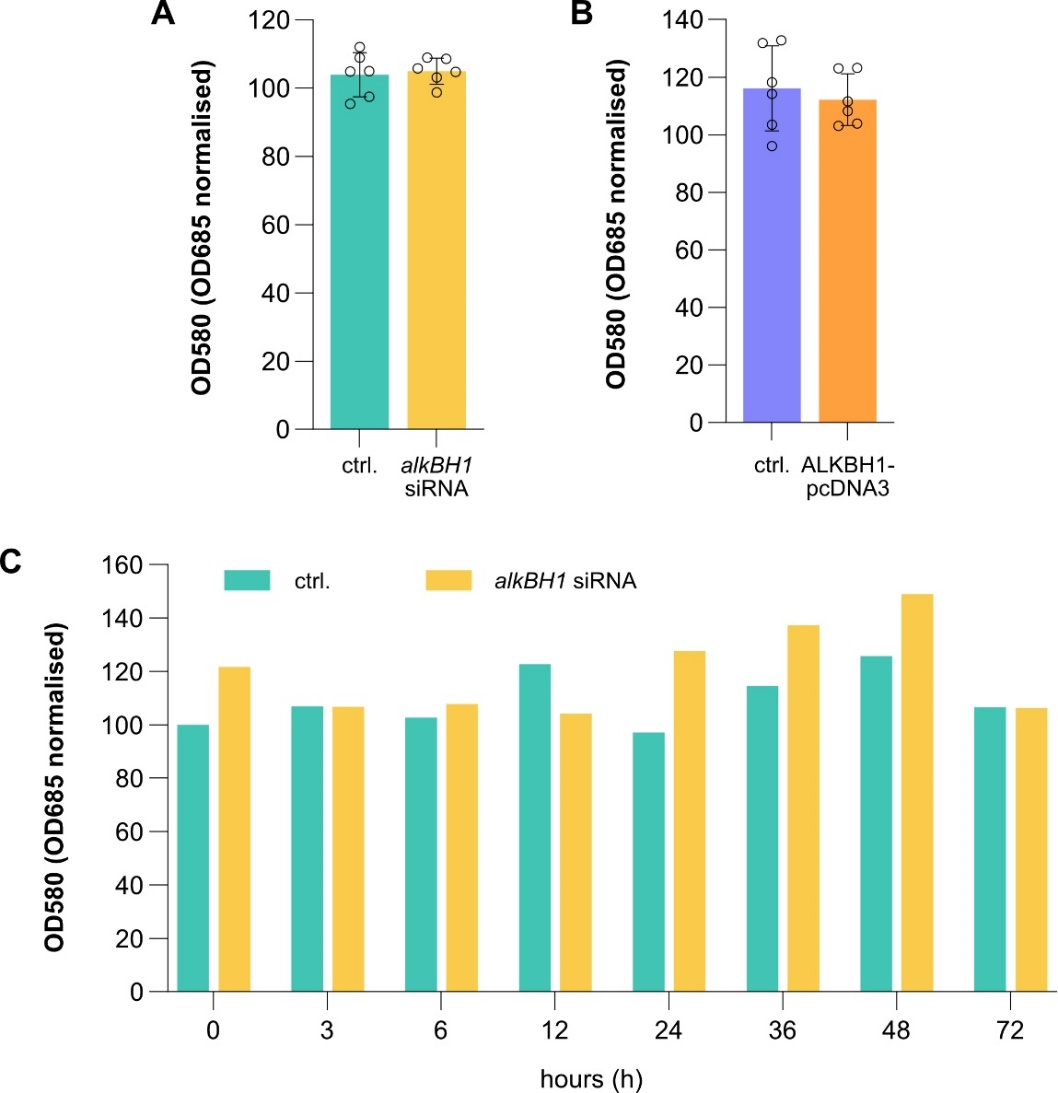

Supplement: S8 Fig — A and B. HEK293T cells were transfected with siRNAs targeting ALKBH1 (Negative control “ctrl”: scrambled siRNA) or ALKBH1-pcDNA3 (Negative control “ctrl”: empty vector) for 48 h. The cell viability was then measured by MTT assay based on the OD value. The mean cell viability was calculated. The cell viability in ctrl. was considered 100% to compare the cell viability in ALKBH1 siRNA or ALKBH1-pcDNA3 transfected cells. C. Cell viability was assessed using the MTT assay, based on OD values for the cycloheximide chase experiment where HEK293T cells were transfected with siRNAs targeting ALKBH1 (Negative control “ctrl”: scrambled siRNA). (TIF) [file pone.0337155.s008.tif]

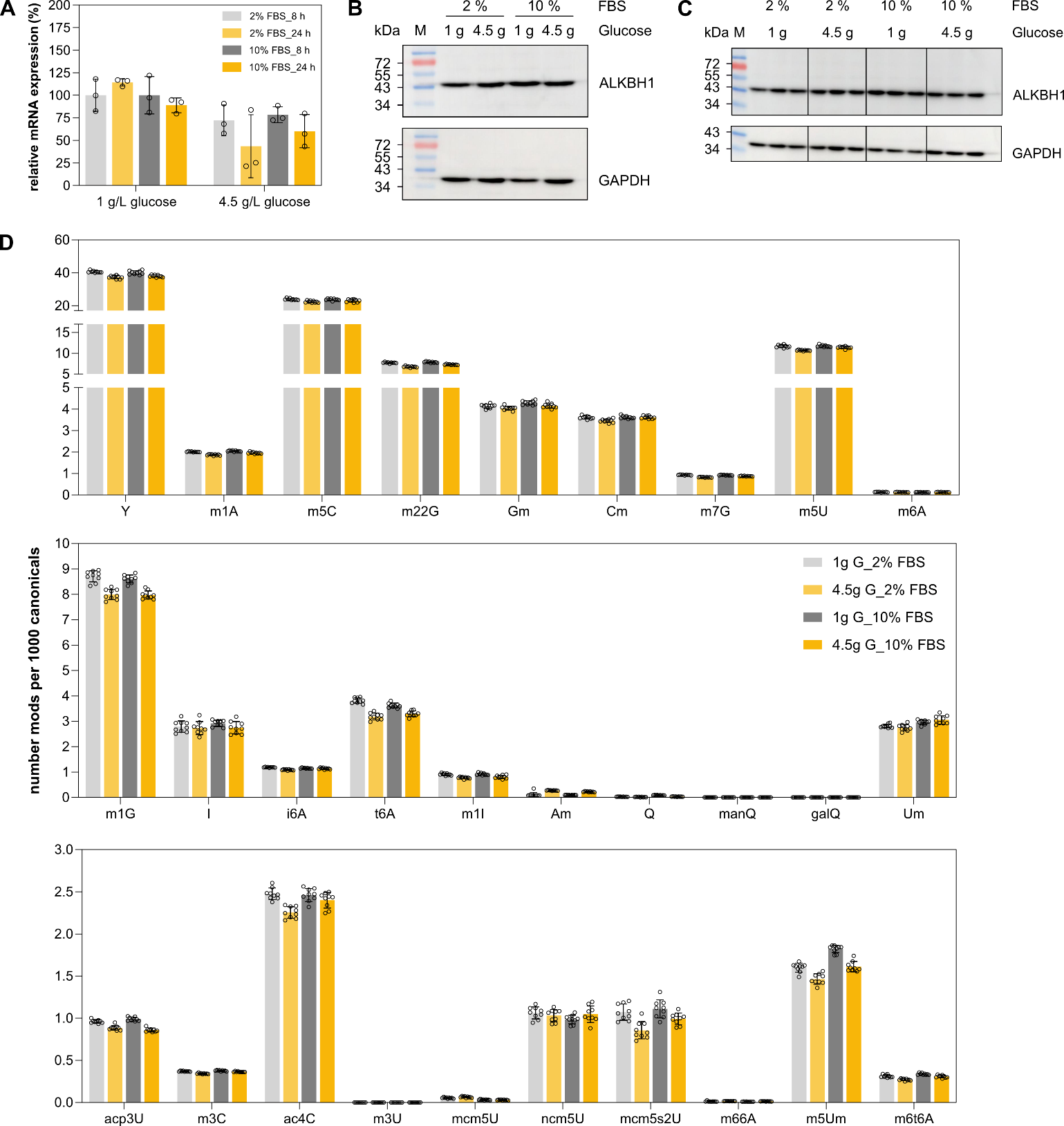

Supplement: S9 Fig — A-C. Longer incubation times (24 h) corresponding to Figure 4. Cells were processed and analyzed by (A) RT-qPCR, and total cell lysates were analyzed by (B; 8 h and C; 24 h) western blotting to assess ALKBH1 expression under varying glucose and serum conditions. D. In parallel, total tRNA was enzymatically digested and subjected to LC-MS/MS for the detection of RNA modifications. Error bars represent the mean ± SEM of three or six biological replicates. Statistical significance was determined using one-way ANOVA without correction. (Created in BioRender. Henzeler, B. (2025).). (TIF) [file pone.0337155.s009.tif]

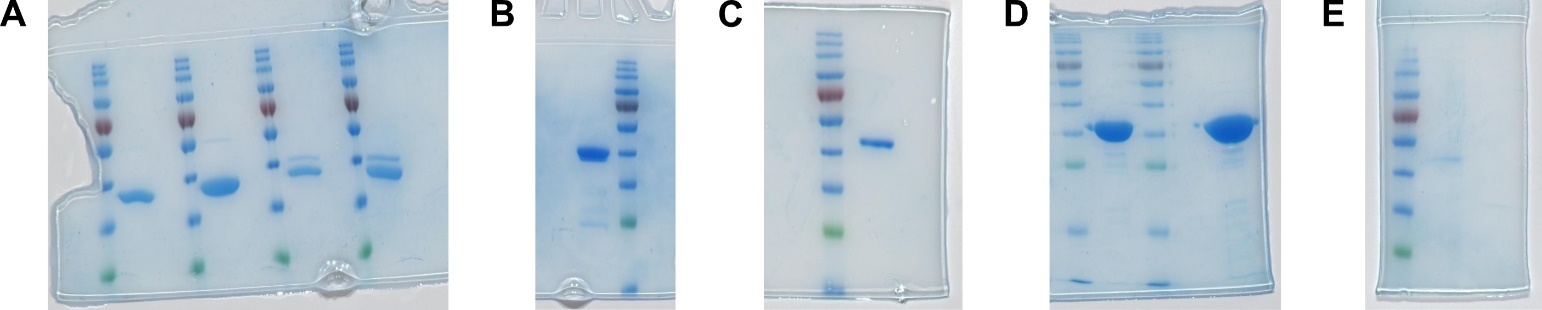

Supplement: S10 Fig — A-D. Human ALKBH1 and ALKBH1I218A, METTL8 and E. coli MiaA were overexpressed and purified from E. coli. E. Human ALKBH1 was overexpressed and purified from HEK293T cells. (TIF) [file pone.0337155.s010.tif]

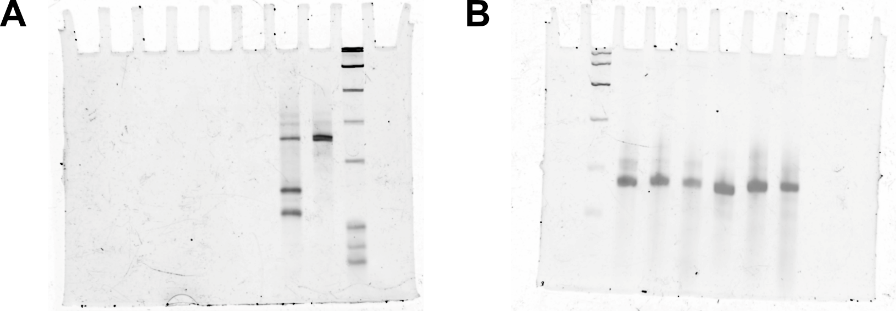

Supplement: S11 Fig — A) shown in Fig 2A and B) Supporting Fig S3. (TIF) [file pone.0337155.s011.tif]

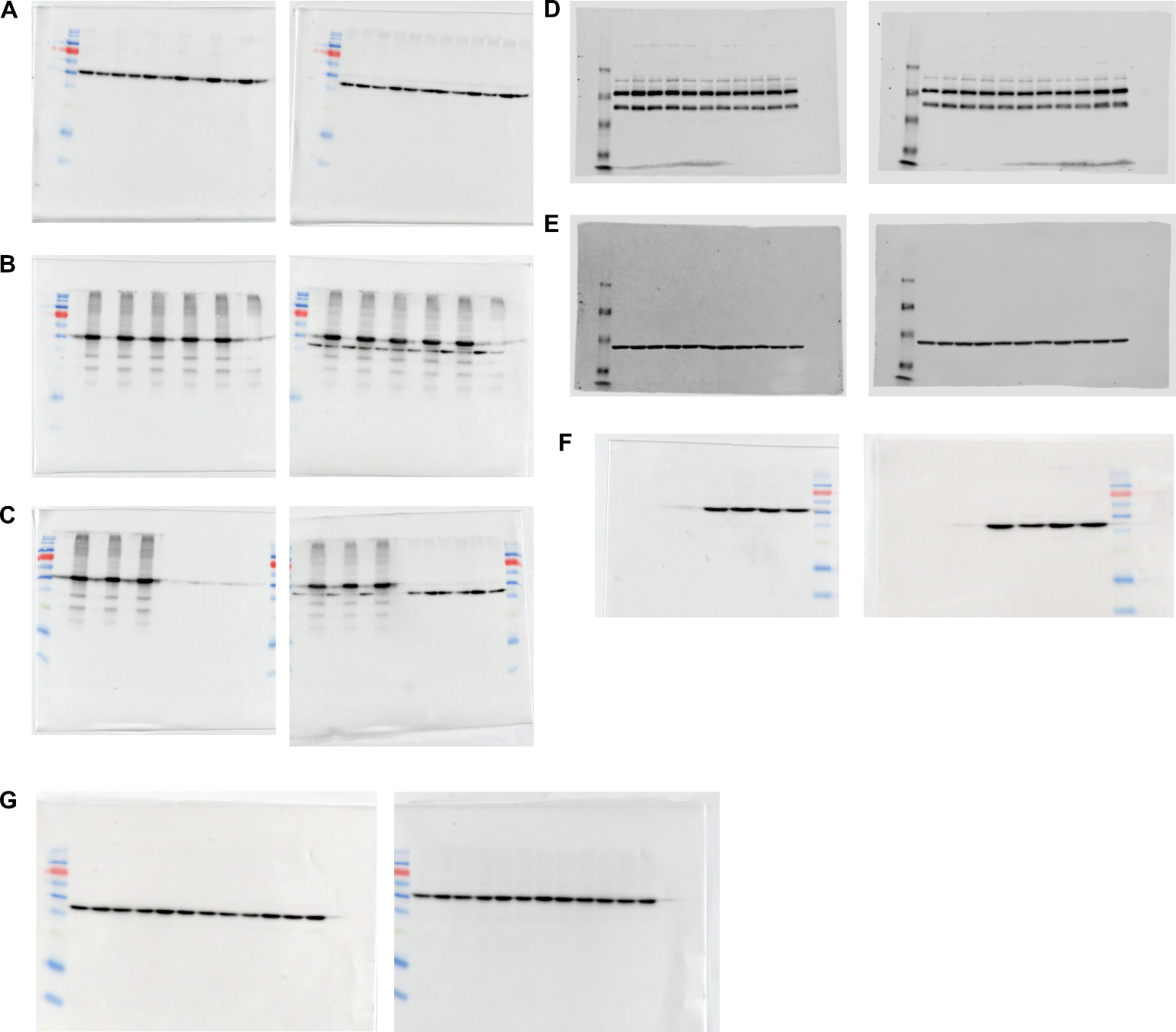

Supplement: S12 Fig — (TIF) [file pone.0337155.s012.tif]
