# Supplementary material for: ALKBH1 activity in vitro and human cell lines by isotope dilution mass spectrometry
Source: PLoS One. 2026 Apr 6;21(4):e0337155. doi: 10.1371/journal.pone.0337155 (PMC13052853; doi:10.1371/journal.pone.0337155)
Supplement: S1 Table — Highlighted in bold and underlined are additional amino acid residues. Ile218 mutated to prevent auto-hydroxylation (ALKBH1I218A) is highlighted in red. His 231 and Asp 233 in the active site, coordinating the iron were mutated to inactivate ALKBH1 (ALKBH1AxA), are highlighted in blue. (PDF) [file pone.0337155.s013.pdf]

Supporting Table S1 Protein sequences. Highlighted in bold and underlined are additional amino acid residues. Ile218 mutated to prevent auto-hydroxylation (ALKBH1^{I218A}) is highlighted in red. His 231 and Asp 233 in the active site, coordinating the iron were mutated to inactivate ALKBH1 (ALKBH1^{AxA}), are highlighted in blue.

**Strep-tagII AlkBH1-19-369 (in pET28) – overexpression in *E. coli* and purification**
**MWSHPQFEK**ENLYFQGNAAGEDAFRKLFRFYRQSRPGTADLEGVIDFSAAHAARGKGPGAQKVIKSQLNVSSVSEQ<br>NAYRAGLQPVSKWQAYGLKGYPGFIFIPNPFLPGYQWHWVKQCLKLYSQKPNVCNLDKHMSKEETQDLWEQSKEFL<br>RYKEATKRRPRSLLEKLRWVTVGYHYNWDSKKYSADHYTPFPSDLGFLSEQVAAACGFEDFRAEAG**I**LNYYRLDST<br>LGI**H**V**D**RSELDHSKPLLSFSFGQSAIFLLGGLQRDEAPTAMFMHSGDIMIMSGFSRLLNHAVPRVLPNPEGEGLPH<br>CLEAPLPAVLPRDSMVEPCSMEDWQVCASYLKTARVNMTVRQVLATDQNFPLEPIEDEKRDISTEG
**AlkBH1-Strep-tagII (in pcDNA3) – overexpression and purification from human cells**
MGKMAAAVGSVATLATEPGEDAFRKLFRFYRQSRPGTADLEGVIDFSAAHAARGKGPGAQKVIKSQLNVSSVSEQNA<br>YRAGLQPVSKWQAYGLKGYPGFIFIPNPFLPGYQWHWVKQCLKLYSQKPNVCNLDKHMSKEETQDLWEQSKEFLRYK<br>EATKRRPRSLLEKLRWVTVGYHYNWDSKKYSADHYTPFPSDLGFLSEQVAAACGFEDFRAEAGILNYYRLDSTLGIH<br>VDRSELDHSKPLLSFSFGQSAIFLLGGLQRDEAPTAMFMHSGDIMIMSGFSRLLNHAVPRVLPNPEGEGLPHCLEAP<br>LPAVLPRDSMVEPCSMEDWQVCASYLKTARVNMTVRQVLATDQNFPLEPIEDEKRDISTEGFCHLDDQNSEVKRARI<br>NPHS**WSHPQFEK**
**hsALKBH1 - full length (in pcDNA3) – overexpression in human cells to investigate effect on tRNA modifications**
MGKMAAAVGSVATLATEPGEDAFRKLFRFYRQSRPGTADLEGVIDFSAAHAARGKGPGAQKVIKSQLNVSSVSEQNA<br>YRAGLQPVSKWQAYGLKGYPGFIFIPNPFLPGYQWHWVKQCLKLYSQKPNVCNLDKHMSKEETQDLWEQSKEFLRYK<br>EATKRRPRSLLEKLRWVTVGYHYNWDSKKYSADHYTPFPSDLGFLSEQVAAACGFEDFRAEAGILNYYRLDSTLGIH<br>VDRSELDHSKPLLSFSFGQSAIFLLGGLQRDEAPTAMFMHSGDIMIMSGFSRLLNHAVPRVLPNPEGEGLPHCLEAP<br>LPAVLPRDSMVEPCSMEDWQVCASYLKTARVNMTVRQVLATDQNFPLEPIEDEKRDISTEGFCHLDDQNSEVKRARI<br>NPHS
**EcMiaA-His$_6$**
MSDISKASLPKAIFLMGPTASGKTALAIELRKILPVELISVDSALIYKGMDIGTAKPNAEELLAAPHRLLDIRDPSQ<br>AYSAADFRRDALAEMADITAAGRIPLLVGGTMLYFKALLEGLSPLPSADPEVRARIEQQAAEQGWESLHRQLQEVDP<br>VAAARIHPNDPQRLSRALEVFFISGKTLTELTQTSGDALPYQVHQFAIAPASRELLHQRIEQRFHQMLASGFEAEVR<br>ALFARGDLHTDLPSIRCVGYRQMWSYLEGEISYDEMVYRGVCATRQLAKRQITWLRGWEGVHWLDSEKP<br>EQARDEVLQVVGAIAG**QHHHHHH**
**METTL8 iso1 Δ1-22-His$_6$**
**MG**SGYHPVAPLGSRILTDPAKVFEHNMWDHMQWSKEEEAAARKKVKENSAVRVLLEEQVKYEREASKYWDTFYKIHK<br>NKFFKDRNWLLREFPEILPVDQKPEEKARESSWDHVKTSATNRFSRMHCPTVPDEKNHYEKSSGSSEGQSKTESDFS<br>NLDSEKHKKGPMETGLFPGSNATFRILEVGCGAGNSVFPILNTLENSPESFLYCCDFASGAVELVKSHSSYRATQCF<br>AFVHDVCDDGLPYPFPDGILDVILLVFVLSSIHPDRMQGVVNRLSKLLKPGGMLLFRDYGRYDKTQLRFKKGHCLSE<br>NFYVRGDGTRAYFFTKGEVHSMFCKASLDEKQNLVDRRLQVNRKKQVKMHRVWIQGKFQKPLHQTQNSSNMVSTLLS<br>QD**LEHHHHHH**
