# Supplementary material for: ALKBH1 activity in vitro and human cell lines by isotope dilution mass spectrometry
Source: PLoS One. 2026 Apr 6;21(4):e0337155. doi: 10.1371/journal.pone.0337155 (PMC13052853; doi:10.1371/journal.pone.0337155)
Supplement: S2 Table — (PDF) [file pone.0337155.s014.pdf]

Supporting Table S2. Used Nucleoside digestion mix.

Component	Stock solution		Target concentration	1x
$MgCl_2$	10 mM	→	1 mM	3 µL
TRIS pH = 8	50 mM	→	5 mM	3 µL
Benzonase	1 U/µL	→	0.8 U	0.8 µL
CIP (Alk.Phos.)	1 U/µL	→	0.8 U	0.8 µL
SPD (PDE1)	0.1 U/µL	→	0.08 U	0.8 µL
Pentostatin	1 mg/mL	→	0,4 µg	0.4 µL
THU	5 mg/mL	→	2 µg	0.4 µL
BHT	10 mM	→	4 µM	0.4 µL
H_2O				0.4 µL
