# Supplementary material for: ALKBH1 activity in vitro and human cell lines by isotope dilution mass spectrometry
Source: PLoS One. 2026 Apr 6;21(4):e0337155. doi: 10.1371/journal.pone.0337155 (PMC13052853; doi:10.1371/journal.pone.0337155)
Supplement: S3 Table — (PDF) [file pone.0337155.s015.pdf]

Supporting Table S3. **tRNA sequences**. Highlighted in bold is the C in the anticodon arm that is methylated by METTL8

**Name**	**Sequence 5' ->3'**
mt-tRNA-Thr^{UGU} "T"	GUCCUUGUAGUAUAAACUAAUACACCAGU**C**UUGUAAACCGGAGAUGAAAACCUUUUUCCAAGGAC
mt-tRNA-Ser^{AGC} "S1"	GAGAAAGCUCACAAGAACUGCUAACUCAUGCCCCCAUGUCUAACAACAUGGCUUUCUCA
mt-tRNA-Ser^{UCA} "S2"	GAAAAAGUCAUGGAGGCCAUGGGGUUGG**C**UUGAAACCAGCUUUGGGGGGUUCGAUUCCUUCCUUUUUUG
ct-tRNA-Val^{AAC}	GUUUCCGUAGUGUAGUGGUUAUCACGUUCGCCUAACACGCGAAAGGUCCCCGGUUCGAAACCGGGCGGAAACACCA
