# Supplementary material for: ALKBH1 activity in vitro and human cell lines by isotope dilution mass spectrometry
Source: PLoS One. 2026 Apr 6;21(4):e0337155. doi: 10.1371/journal.pone.0337155 (PMC13052853; doi:10.1371/journal.pone.0337155)
Supplement: S4 Table — (PDF) [file pone.0337155.s016.pdf]

Supporting Table S4. qPCR primers

alkB-strep_F	agtttgaaaaatgaGCATGCATCTAGAGGGCC
alkB_R	gcggatggctccaGCTGTGAGGGTTTATCCTG
EcMiaA_F	catgCCATGggcAGTGATATCAGTAAGGCGAGCCTG
EcMiaA_R	ccgCTCGAGgcctgcgatagcaccaacaac
AlkBH1_F	TTATCCCAAACCCCTTCCTC
AlkBH1_R	GTCTCCGTTTAGTCGCTTC
actin_F	GCTAAGTCCTGCCCTCATTT
actin_R	GTACAGGTCTTTGCGGATGT
18srRNA_F	CACGGACAGGATTGACAGATT
18srRNA_R	GCCAGAGTCTCGTTCGTTATC
