# Supplementary material for: ALKBH1 activity in vitro and human cell lines by isotope dilution mass spectrometry
Source: PLoS One. 2026 Apr 6;21(4):e0337155. doi: 10.1371/journal.pone.0337155 (PMC13052853; doi:10.1371/journal.pone.0337155)
Supplement: S5 Table — (PDF) [file pone.0337155.s017.pdf]

Supporting Table S5. siRNA sequences

Silencer select siRNA for hALKBH1	ThermoFisher, AM16704, Assay ID 107924
Silencer select siRNA for hALKBH1	ThermoFisher, AM16704, Assay ID 17144
siRNA for ALKBH1	Sigma Aldrich, EHU135521-50µg
Silencer™ Negativkontrolle Nr. 1 siRNA	ThermoFisher, AM4635
Mission esiRNAs	Sigma Aldrich, EHUFLUC-50µg
