# Supplementary material for: ALKBH1 activity in vitro and human cell lines by isotope dilution mass spectrometry
Source: PLoS One. 2026 Apr 6;21(4):e0337155. doi: 10.1371/journal.pone.0337155 (PMC13052853; doi:10.1371/journal.pone.0337155)
Supplement: S6 Table — (PDF) [file pone.0337155.s018.pdf]

Supporting Table S6. Biotinylated capture probes for tRNA molecules

mt-tRNA-Thr^{UGU}	5'-[Biotin]-TGTCCTTGGAAAAAGGTTTTCATCTCCGGTaaa-3'
mt-tRNA-Ser^{AGC}	5'-[Biotin]- TGGTGAGAAAGCCATGTTGTTAGACATGGGaaa-3'
mt-tRNA-Ser^{UCA}	5'-[Biotin]- ACAAAAAAGGAAGGAATCGAACCCCCCAAAaaa-3'
