# Supplementary material for: ALKBH1 activity in vitro and human cell lines by isotope dilution mass spectrometry
Source: PLoS One. 2026 Apr 6;21(4):e0337155. doi: 10.1371/journal.pone.0337155 (PMC13052853; doi:10.1371/journal.pone.0337155)
Supplement: S7 Table — (PDF) [file pone.0337155.s019.pdf]

Supporting Table S7. Antibodies used in this study

Recombinant Anti-ALKBH1 antibody [EPR6176]	abcam, ab126596
beta Actin Monoclonal Antibody (15G5A11/E2)	ThermoFisher Sci. MA1-140
GAPDH Rabbit Monoclonal Antibody (14C10)	Cell Signaling, 2118S
