# Supplementary material for: ALKBH1 activity in vitro and human cell lines by isotope dilution mass spectrometry
Source: PLoS One. 2026 Apr 6;21(4):e0337155. doi: 10.1371/journal.pone.0337155 (PMC13052853; doi:10.1371/journal.pone.0337155)
Supplement: S8 Table — (PDF) [file pone.0337155.s020.pdf]

Supporting Table S8. Abbreviations of the specified RNA modifications are listed below

**Abbreviation**	**Modification**
Y	Pseudouridine
D	Dihydrouridine
m^2G	N2-methylguanosine
m^1A	N1-methyladenosine
m^5C	5-methylcytidine
$m^{22}G$	N2,N2-dimethylguanosine
Gm	2'-O-methylguanosine
Cm	2'-O-methylcytidine
m^7G	7-methylguanosine
m^5U	5-methyluridine
m^6A	N6-methyladenosine
m^1G	N1-methylguanosine
I	Inosine
i^6A	N6-isopentenyladenosine
t^6A	N6-threonylcarbamoyladenosine
m^1I	N1-methylinosine
Am	2'-O-methyladenosine
Q	Queuosine
ManQ	Mannosyl-queuosine
GalQ	Galactosyl-queuosine
Um	2'-O-methyluridine
acp^3U	3-(3-amino-3-carboxypropyl)uridine
m^3C	3-methylcytidine
ac^4C	N4-acetylcytidine
m^3U	3-methyluridine
mcm^5U	5-methoxycarbonylmethyluridine
ncm^5U	5-carbamoylmethyluridine
mcm^5s^2U	5-methoxycarbonylmethyl-2-thiouridine
$m^{6},^{6}A$	N6,N6-dimethyladenosine
m^5Um	5-methyl-2'-O-methyluridine
m^6t^6A	N6-threonylcarbamoyl-N6-methyladenosine
ms^2i^6A	2-methylthio-N6-isopentenyladenosine
