# Supplementary material for: ALKBH1 activity in vitro and human cell lines by isotope dilution mass spectrometry
Source: PLoS One. 2026 Apr 6;21(4):e0337155. doi: 10.1371/journal.pone.0337155 (PMC13052853; doi:10.1371/journal.pone.0337155)
Supplement: Attachment — Submitted filename: response-to-reviewer.docx [file pone.0337155.s022.docx]

We thank again the reviewer for their time and thoughtful comments that have allowed us to further improve our manuscript.

**Comments to the Author**

1. If the authors have adequately addressed your comments raised in a previous round of review and you feel that this manuscript is now acceptable for publication, you may indicate that here to bypass the “Comments to the Author” section, enter your conflict of interest statement in the “Confidential to Editor” section, and submit your "Accept" recommendation.

Reviewer #1: All comments have been addressed

Reviewer #2: All comments have been addressed

2. Is the manuscript technically sound, and do the data support the conclusions?

The manuscript must describe a technically sound piece of scientific research with data that supports the conclusions. Experiments must have been conducted rigorously, with appropriate controls, replication, and sample sizes. The conclusions must be drawn appropriately based on the data presented.

Reviewer #1: Yes

Reviewer #2: Yes

3. Has the statistical analysis been performed appropriately and rigorously?

Reviewer #1: Yes

Reviewer #2: Yes

4. Have the authors made all data underlying the findings in their manuscript fully available?

The [PLOS Data policy](http://track.editorialmanager.com/CL0/http:%2F%2Fwww.plosone.org%2Fstatic%2Fpolicies.action%23sharing/1/010f019c407e7223-5f85e055-6cd8-4e9e-894f-5d24cb8ddc79-000000/8B87FBwpZLoYzKHGPTnCQoDOSrw9sg5El5_wg1Cgz7Y=249) requires authors to make all data underlying the findings described in their manuscript fully available without restriction, with rare exception (please refer to the Data Availability Statement in the manuscript PDF file). The data should be provided as part of the manuscript or its supporting information, or deposited to a public repository. For example, in addition to summary statistics, the data points behind means, medians and variance measures should be available. If there are restrictions on publicly sharing data—e.g. participant privacy or use of data from a third party—those must be specified.

Reviewer #1: Yes

Reviewer #2: Yes

5. Is the manuscript presented in an intelligible fashion and written in standard English?

PLOS ONE does not copyedit accepted manuscripts, so the language in submitted articles must be clear, correct, and unambiguous. Any typographical or grammatical errors should be corrected at revision, so please note any specific errors here.

Reviewer #1: Yes

Reviewer #2: Yes

6. Review Comments to the Author

Please use the space provided to explain your answers to the questions above. You may also include additional comments for the author, including concerns about dual publication, research ethics, or publication ethics. (Please upload your review as an attachment if it exceeds 20,000 characters)

Reviewer #1: The authors responded to my comments in an excellent manner. I agree with their assessment of ALKBH1 function, especially in light of new evidence. I have 2 minor comments:
1- IN Nakayashiki et al's preprint, they used 4 cell lines and not 5 (This was a typo in my previous comment, but the authors should double check)

In the cited manuscript by Nakayashiki et al 5 cell lines were used. However, 4 are only relevant for our manuscript. Thus we have corrected it in the manuscript.

2- The authors should add a limitation section in their discussion including some of the points they discussed (mitochondrial tRNA limitations, technology limitations in assessing other modifications, different results compared to literature due to different approaches, etc.)
“In this context quantitative LC-MS analysis must be considered the gold standard in nucleoside detection due to its high specificity and sensitivity, that other detection methods such as dot blots cannot provide. …. ***Nevertheless, a main challenge for detecting and quantifying modified ribonucleosides is the reproducibility across laboratories [40]. Here variations in growth conditions, cell lines, RNA isolation and processing as well as lack of a standard form of modified RNA for researchers to use as a benchmark for validating their studies hamper the accurate quantification of RNA modifications. In addition, there is no single optimal method for all 170 known modified ribonucleosides or the more than 50 human RNA modification.****”*

Apart from those 2 minor points, I believe my concerns and comments are all addressed.

We thank the reviewer for his time and valuable comments.

Reviewer #2: In this revision, the authors strengthen their manuscript by (1) adding protein-level validation of ALKBH1 knockdown/overexpression and documenting an unusually long ALKBH1 half-life, (2) extending glucose/FBS stress experiments to 24 h with matched ALKBH1 mRNA/protein and tRNA nucleoside LC–MS/MS readouts, (3) adding a –α-KG control and rewording prior “Fe(II)/α-KG dependency” language, and (4) providing DLS evidence supporting in vitro tRNA refolding. In summary, these additional experiments has addressed my previous concerns and improved the rigor of this study. They also appropriately narrow several over-scoped claims. The paper is suitable for publication.

We thank the reviewer for his time and valuable comments.
